# Assessment of Microstressors in Adults: Questionnaire Development and Ecological Validation of the Mainz Inventory of Microstressors

**DOI:** 10.2196/14566

**Published:** 2020-02-24

**Authors:** Andrea Chmitorz, Karolina Kurth, Lara K Mey, Mario Wenzel, Klaus Lieb, Oliver Tüscher, Thomas Kubiak, Raffael Kalisch

**Affiliations:** 1 Department of Psychiatry and Psychotherapy University Medical Center Mainz Mainz Germany; 2 Faculty of Social Work, Health Care and Nursing Sciences Esslingen University of Applied Sciences Esslingen Germany; 3 Health Psychology Institute for Psychology Johannes Gutenberg University Mainz Germany; 4 Leibniz Institute for Resilience Research Mainz Germany; 5 Neuroimaging Center University Medical Center Mainz Germany

**Keywords:** microstressor, daily hassles, validation, ecological momentary assessment

## Abstract

**Background:**

Many existing scales for microstressor assessment do not differentiate between objective (ie, observable) stressor events and stressful cognitions or concerns. They often mix items assessing objective stressor events with items measuring other aspects of stress, such as perceived stressor severity, the evoked stress reaction, or further consequences on health, which may result in spurious associations in studies that include other questionnaires that measure such constructs. Most scales were developed several decades ago; therefore, modern life stressors may not be represented. Ecological momentary assessment (EMA) allows for sampling of current behaviors and experiences in real time and in the natural habitat, thereby maximizing the generalization of the findings to real-life situations (ie, ecological validity) and minimizing recall bias. However, it has not been used for the validation of microstressor questionnaires so far.

**Objective:**

The aim is to develop a questionnaire that (1) allows for retrospective assessment of microstressors over one week, (2) focuses on objective (ie, observable) microstressors, (3) includes stressors of modern life, and (4) separates stressor occurrence from perceived stressor severity.

**Methods:**

Cross-sectional (N=108) and longitudinal studies (N=10 and N=70) were conducted to evaluate the Mainz Inventory of Microstressors (MIMIS). In the longitudinal studies, EMA was used to compare stressor data, which was collected five times per day for 7 or 30 days with retrospective reports (end-of-day, end-of-week). Pearson correlations and multilevel modeling were used in the analyses.

**Results:**

High correlations were found between end-of-week, end-of-day, and EMA data for microstressor occurrence (counts) (*r*≥.69 for comparisons per week, *r*≥.83 for cumulated data) and for mean perceived microstressor severity (*r*≥.74 for comparisons per week, *r*≥.85 for cumulated data). The end-of-week questionnaire predicted the EMA assessments sufficiently (counts: beta=.03, 95% CI .02-.03, *P*<.001; severity: beta=.73, 95% CI .59-.88, *P*<.001) and the association did not change significantly over four subsequent weeks.

**Conclusions:**

Our results provide evidence for the ecological validity of the MIMIS questionnaire.

## Introduction

### Background

The impact of microstressors on mental health, either alone or in addition to macrostressors, has been reported in a large body of research [1-5]. The term *macrostressor* refers to potentially traumatizing events, such as natural or human-made disasters, whereas the term *microstressor*, or daily hassle, refers to the “irritating, frustrating, distressing demands that to some degree characterize everyday transactions with the environment” ([4] page 3).

For the assessment of stress and stressors, several approaches have been suggested and discussed in the literature. The response-based approach focuses on the effect of stressors on the individual. This line of research emerged with Selye [6], who was particularly interested in the physiological response to stress and the development of illness. However, it has been criticized that the response-based approach does not take into account the characteristics of the stressor, but rather assumes a nonspecific response to adverse stimulations regardless of the situation [7]. Instead of focusing on the individual response to stress, the stimulus-based approach suggests focusing on the stressor itself. This approach has its origins in the work by Holmes and Rahe [8], who measured life stress by assigning numbers (so-called *life change units*) to a list of critical life events to assess the adaptive effort required to cope with the event. The stimulus-based approach has also been applied to assess the effect of microstressors [9]. Stone and Shiffman [10] pointed out that the frequency and type of stressors occurring in a certain time period provide information about the level of stress experienced in the same period.

### Assessment of Microstressors

So far, a number of validated self-report scales for the assessment of microstressors have been developed. The first validated scales for the assessment of microstressors are the Hassles and Uplifts Scales [4,11]. Several other microstressor questionnaires have been published subsequently, such as the Inventory of Small Life Events [12], the Daily Stress Inventory [13], and the Weekly Hassle Scale [14]. Moreover, questionnaires for specific target groups have been published, such as the Adolescent Stress Questionnaire [15], an adaptation of the Everyday Stressor Index [16] for the assessment of microstressors occurring in everyday life of Turkish or German mothers with young children, or a microstressor questionnaire for students, the Inventory of College Students’ Recent Life Experiences (ICSRLE) [17]. Multimedia Appendix 1 provides an overview of the questionnaires.

### Methodological Considerations in the Assessment of Microstressors

A criticism is that many of the existing microstressor scales do not exclusively focus on objective (ie, observable) stressors, but also include items assessing cognitions, emotions, and consequences of stress or symptoms, which may conceptually overlap other questionnaires assessing the same constructs and may consequently result in spurious associations [5,7,14,18-21]. In clinical routine, the issue of nonobservable stressors and spurious associations may be negligible when assessing patients on an individual level to obtain information on their current stressor load; however, the methodological issue arises in studies on associations between microstressors and other topics or concepts that are also partially covered by items in the microstressor questionnaire. For example, the Daily Hassles and Uplifts Scale includes items about inner concerns (eg, trouble making decisions or concern about the meaning of life) [11], and similar items may also be found in symptom scales of stress-related mental disorders. Consequently, in studies using both scales, the overlapping items and constructs may result in an overestimation of the association between the hassles scale and the symptom scale. This may lead to wrong conclusions about the impact of microstressors on mental health because similar questions were asked in both questionnaires. In resilience research, for example, it is theorized that individual differences in the subjective reactions to stressors are a key determinant of why some people stay healthy under stressor exposure while others with similar stressor exposure develop mental health problems [22]. This theory can obviously only be tested if one can separately quantify stressor exposure and subjective reactions to the stressor exposure.

To avoid this methodological issue, it has been suggested to strictly focus on objective (ie, observable) situations instead of subjective aspects, such as interpretations, cognitions, emotions, or symptoms [5,18,21]. This allows for an unconfounded analysis of the effect of microstressors on the outcome in question (eg, perceived distress or physical health). In some studies, this issue is addressed by excluding potentially confounding items [23]. Until now, there have been only a few microstressor questionnaires in which that issue has been taken into account during the development phase of the questionnaire [12].

Many of the existing questionnaires were developed and validated between 1980 and 1990 [4,11-13]. Consequently, stressors that have occurred as a consequence of later developments, such as globalization, urbanization, and digitalization, may not be represented.

All studies validating the previously mentioned questionnaires rely on retrospective data. Real-time data, as obtained by using ecological momentary assessment (EMA), has rarely been used for the validation of microstressor questionnaires. EMA methods allow for sampling of current behaviors and experiences of a subject in real time and in their natural habitat [24]. EMA aims to maximize ecological validity (ie, generalization of the findings to real-life situations) to minimize recall bias, and it also allows for the study of microprocesses that impact behavior in real-world contexts [24]. The method has already been applied in studies on the effects of microstressors [25-27] and for comparisons between retrospective and momentary data for alcohol consumption [28], headache [29], pain [30], or affect and sexual behavior [31], for example. A recent systematic review evaluated studies on mobile phone-based self-assessment of stress in healthy adults [32]. The authors found in only three of 35 studies included in the review was the validity of the mobile phone-based stress assessment against validated retrospective stress questionnaires examined [33-35]. In one of those studies (N=48 participants), a moderate statistically significant positive correlation was found (*r*=.4, *P*<.05) [35]. No statistically significant correlations were found in the other two studies, which may be caused by small sample sizes (N=7 and N=17) [33,34]. Pórarinsdóttir and colleagues [36] also conducted a study and found a statistically significant positive correlation between mobile phone-based stress assessment and a validated stress scale (beta=.0167, 95% CI .0070-.0026; *P*=.001). However, all four studies used the Cohen’s Perceived Stress Scale [37] for validation of the mobile phone-based stress assessment, which focuses on subjective, rather than objective, aspects of stress.

### Aims and Objectives of This Study

In this study, we aimed to develop a questionnaire that (1) allows for retrospective assessment of microstressors occurring during the course of one week, (2) focuses on objective (ie, observable) microstressors to overcome the risk of spurious associations caused by assessing subjective aspects (such as cognitions or emotions), (3) also includes stressors of modern life, and (4) combines the stimulus- and response-based approach for stressor assessment to measure the occurrence of the stressors and the perceived severity of the stressor.

In addition, we applied a validation strategy that maximizes ecological validity by using EMA for the validation of the questionnaire.

## Methods

### Overview

The validation of the questionnaire was conducted in three phases. The first phase involved item generation of objective stressors (see Multimedia Appendix 2), the second phase involved questionnaire construction and revision, and the third phase involved EMA evaluation of the retrospective questionnaire. The first version of the questionnaire included 67 items and was applied in the first and the second phase of questionnaire development. The final version of the questionnaire included 58 items and was used in the third phase. In both versions of the questionnaire, participants were asked to provide information about microstressors occurring during the past seven days. To obtain additional information about the individual impact of each stressor, the questionnaire includes a five-point Likert scale (0-4; 0=not at all severe, 4=extremely severe) after each item, asking for the perceived severity of the stressor (see Multimedia Appendix 2).

All participants were recruited at the Johannes Gutenberg University, Mainz, Germany. Data were collected online using the survey tool SoSci Survey [38].

The study protocols were approved by the ethics committee at the Rhineland-Palatinate state chamber of physicians (837.085.13 [8770-F] and 837.183.16 [10502]). The Checklist for Reporting Results of Internet E-Surveys (CHERRIES) [39] was applied (see Multimedia Appendix 3).

### Questionnaire Construction and Revision

A cross-sectional study (study 1) and a small-scale EMA feasibility study (study 2) were conducted to evaluate the 67-item version of the Mainz Inventory of Microstressors (MIMIS) (study 1) and to test the feasibility of a mobile phone-based EMA assessment of the questionnaire (study 2).

### Study 1: Cross-Sectional Study

There were no explicit inclusion or exclusion criteria. The sample included 120 undergraduate students (data collection period: October 2014 to January 2016). All participants completed a questionnaire assessing sociodemographic variables (age and sex) and the 67-item version of the MIMIS, retrospectively assessing the number of microstressors and their severity over the past seven days. A free-text input was provided to include additional microstressors in case the experienced microstressor was not already on the list. Data from 108 participants were used for the assessment of the questionnaire; 11 participants did not complete the questionnaire and one participant was excluded due to extreme response tendency (ie, all items rated at the highest level). The final sample included 72.5% women (79/108) and 27.5% men (30/108). The mean age was 23.91 (SD 4.06, range 18-43) years.

### Study 2: Ecological Momentary Assessment Feasibility

This study was conducted to test the feasibility of a mobile phone-based EMA assessment of the questionnaire. Inclusion criteria were no severe mental disorder (eg, schizophrenia) and good mental health (screening questionnaire: General Health Questionnaire total score <24 [40]). Participants who were in current psychiatric or psychotherapeutic treatment and users of illegal drugs or those with reported high levels of alcohol consumption (average consumption of standard glasses of alcohol per week >15) were excluded [41,42]. Potential participants were invited for an initial briefing session. After written consent was obtained, each participant was provided with a study mobile phone (type: Motorola Moto E) to avoid technical problems related to different operating systems. The study was conducted over seven subsequent days.

For the mobile phone-based EMA assessment, we implemented the 67 items of the MIMIS questionnaire in a mobile phone-based ambulatory assessment using the app MovisensXS [43]. Ambulatory data of microstressors were collected with an event-contingent assessment (ie, participants recorded the microstressor immediately after it occurred). After activating the MovisensXS app, participants were asked to select from the list of 67 prespecified microstressors. In case the experienced microstressor was not already in the list, a free-text input was provided to include additional microstressors. As in the original version of the MIMIS (end-of-week assessment, see study 1), participants were asked to rate the severity of the selected microstressor. Data entry was possible at any time. In addition to the mobile phone-based assessment, we administered a modified version of the MIMIS at the end of each day, asking for microstressors occurring on that particular day (“How many times did the situation occur during the day?” “To what extent did you find the situations mentally straining?”). At the end of the seven-day assessment period, we also used the original version of the 67-item MIMIS questionnaire (see study 1). The data collection ended with a final session the following week. At this final session, participants returned the study mobile phones and were asked to provide feedback on the study in a semistructured interview.

The sample included 10 undergraduate students (six females; data collection: June 2016). The mean age of participants was 26.6 (SD 2.05, range 23-30) years.

The data from study 1 and study 2 were used to revise the 67-item questionnaire. We analyzed the data of both studies (1 and 2) by considering the total occurrence per microstressor, and excluded those microstressors that were discarded (ie, frequency=0; 19 items removed). We used the information provided by the free-text input to identify additional relevant microstressors, which resulted in 10 additional items. We also revised the wording of the items to emphasize the objective character of the microstressor. The revised version of the MIMIS questionnaire consisted of 58 items covering a large range of aspects of daily living (eg, noise, traffic, interpersonal conflicts, workload or time pressure) (see Multimedia Appendix 4). The 58-item questionnaire was then included in a four-week longitudinal EMA study using the study design tested in study 2.

### Study 3: Longitudinal Ecological Momentary Assessment Study

#### Sample

**Figure 1:**
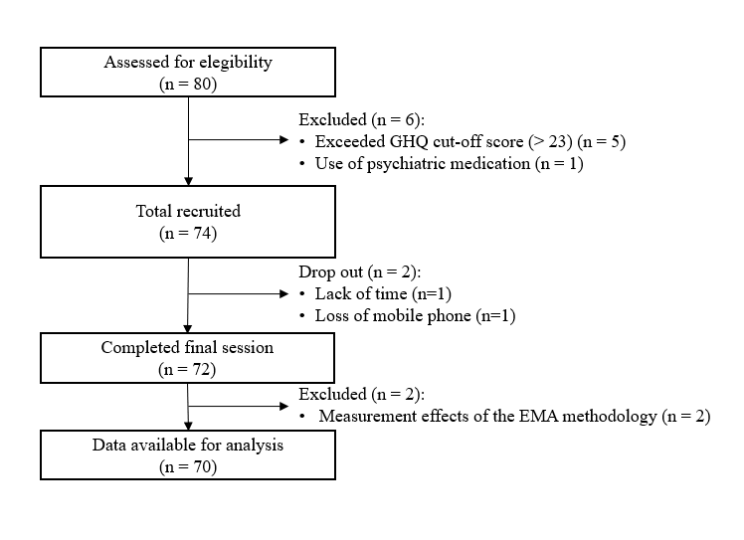
Flowchart of the longitudinal ecological momentary assessment study (study 3).

Data collection was between September 2016 and March 2017. We applied the same exclusion criteria as in study 2. Figure 1 provides an overview of the recruitment process. Two participants reported changes in their behavior (handling of microstressors) during the study period because of the EMA assessments in the postmonitoring interview. Therefore, we excluded the data after data collection. The final sample included 70 participants with a mean age of 23.93 (SD 3.15) years.

### Study Design and Procedures

For each participant, the study was conducted over four subsequent weeks (28 days). We used the same procedure as in study 2. In addition, data on sociodemographic variables (sex, age, employment status, nationality, and education), mental health-related variables (mental dysfunction and well-being), and chronic stress (discussed subsequently) were collected. The revised 58-item version of the MIMIS questionnaire was implemented in a mobile phone-based ambulatory assessment. In contrast to study 2, we used a signal-contingent approach; that is, an acoustic and visual signal (“please answer the questions below” on the display) notifying participants to record data on the occurrence and perceived severity of microstressors at five random time points between 9 am and 8 pm for 28 subsequent days. If participants were not able to answer the questionnaire by the time the signal occurred, they were reminded every 30 minutes to complete the questionnaire for the subsequent 90 minutes. Participants could also ignore the initial signal and manually activate data entry during the following 90 minutes. As in study 2, at each assessment point, participants were provided with the list of microstressors and asked whether any of these occurred since the last alarm (“Please indicate which of the following situations occurred since the last alarm, independent from whether they were perceived as a hassle or not”). For each selected microstressor, participants were then asked to rate the severity of the stressor on a five-point Likert scale from 0 (not at all severe) to 4 (very severe). Similar to study 2, participants completed an end-of-day assessment on each of the 28 days and an end-of-week assessment at the end of each of the four weeks using the 58-item MIMIS questionnaire with the respective time scale (past day, past seven days). Every evening at 7:58 pm or every Sunday at 11 am, participants were reminded via email with a link to the online survey to complete the end-of-day or end-of-week assessment, respectively. As in study 2, a final session was conducted in the week after the 28-day assessment period, in which participants returned the study mobile phones and provided feedback in a semistructured interview.

**Figure 2:**
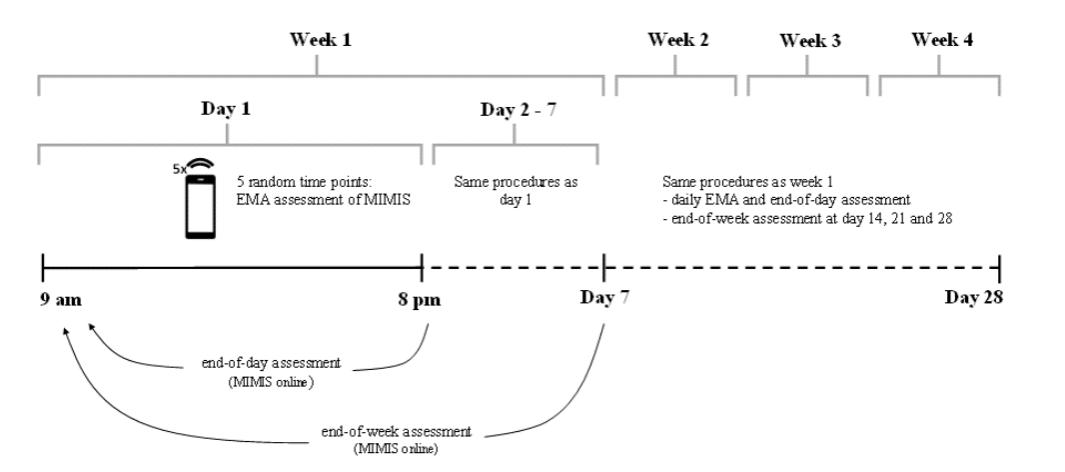
Study design of the longitudinal ecological momentary assessment study.

The participants received monetary compensation at the end of the study. Here we applied a scoring system to increase the motivation to participate in the study and provide complete datasets. The score accounted for the number of complete datasets provided in the EMA assessments and the online questionnaires. Participants received up to €176 (see Multimedia Appendix 3). To increase compliance, participants were informed of their actual total score every evening via email during the 28-day study period. Figure 2 provides an overview of the study design.

### Statistical Analysis

#### Sample Characteristics

To describe the sample, proportions were derived for categorical variables; means and standard deviations were used for continuous variables.

#### Quantification of Retrospective Bias

The primary analysis was the analysis of the retrospective bias of the MIMIS questionnaire assessing microstressors at the end of a week over the past seven days.

To examine the level of retrospective bias of the MIMIS, we compared the EMA, end-of-day, and end-of-week assessments for each of the four weeks and over the entire four-week period using Pearson correlations. Here, we considered the total counts (number of days on which the microstressor occurred) of all 58 microstressors. To reach comparability with the end-of-week assessment, microstressors measured with the EMA and end-of-day assessments were counted nominally (0=did not occur during the day, 1=did occur at least one time during the day) per day. For the correlations of severity, we considered the mean of the average severity of each microstressor over all microstressors in the end-of-week, end-of-day, and EMA assessments. In addition, we used multilevel modeling to further assess to what extent the end-of-week assessment predicted the ecologically valid EMA assessments and whether the association varied over the four subsequent weeks. Two models were applied: model 1 included the total counts and model 2 included the mean severity ratings of all microstressors reported in the end-of-week assessment or EMA. The outcome was the end-of-week assessment; level one was the observations in EMA (model 1: total counts; model 2: mean stressor severity), level two was the weeks, and level three was the participants. We first calculated the null model to assess whether there was an intraclass correlation (ICC), which refers to the extent of variance that can be explained by differences within and between persons. We subsequently included several predictors hierarchically, including total counts of all microstressors of each week (model 1) or mean severity over all microstressors of each week (model 2), age, sex, week of assessment, and the interaction term total counts of microstressors assessed in EMA × week of assessment, and analyzed whether model fit was improved by the predictors using likelihood ratio tests. We used likelihood ratio tests to determine whether the predictors should not only be included as fixed effects but also as random effects. This would allow for the slopes of the association between the predictors and the criterion to vary between the participants [44]. Model 1 included the interaction term “total counts of microstressors assessed in EMA × week of assessment” as a fixed effect and “week of assessment and total counts of all microstressors of each week” as a random effect as indicated by likelihood ratio tests. Model 2 included the interaction term “mean severity over all microstressors of each week × week of assessment” and age as fixed effects and week of assessment as a random effect, as indicated by likelihood ratio tests.

Statistical significance of effects was determined by *P* values of less than .05 or by 95% confidence intervals (CIs). All analyses were conducted in Stata version 15 [45].

## Results

### Sample Characteristics

Table 1 provides an overview of the sample characteristics in the longitudinal EMA study. Of the 70 participants, 41 (59%) were women; 66 of 70 (94%) were German and 47 of 70 (67%) worked 20 hours or less per week. The study adherence was excellent. On average, the participants completed 90% of the assessments (end-of-day, end-of-week, and EMA) over the four weeks.

**Table 1:** Descriptive statistics of the psychometric study sample (N=70).^a^

Variable		Participants
**Gender, n (%)**		
	Female	41 (59)
	Male	29 (41)
Age (years), mean (SD)		23.9 (3.2)
**Nationality, n (%)**		
	German	66 (94)
	Others	4 (6)
**Employment status, n (%)**		
	Full-time	5 (7)
	Part-time^b^	15 (21)
	Others^c^	32 (46)
	Not employed	18 (26)

^a^All participants had a high school diploma (≥12 years of formal education) or equivalent.

^b^18-20 hours per week.

^c^Occasional jobs, jobs with less than 18 hours per week.

In total, the participants responded to 9162 of the EMA prompts and missed 478 prompts. They filled in 1935 end-of-day and 282 end-of-week assessments. We excluded 39 end-of-day forms and 6 end-of-week assessments because the questionnaires were not filled in within the prescribed time period (end-of-day: n=29, end-of-week: n=3) or were submitted twice (end-of-day: n=10, end-of-week: n=3). Participants missed 64 end-of-day assessments and 4 end-of-week assessments. With regard to stressor frequency, the 10 most frequent (in counts) stressors reported in the end-of-week assessment were journey/commute to work, university, or school (n=65, counts: 987); housekeeping (n=66, counts: 982); waiting time or delay (n=68, counts: 741); interruption during an activity (n=62, counts: 693); high demands or high workload at work, school, or university (n=54, counts: 670); time pressure (n=59, counts: 666); lack of sleep (n=66, counts: 603); own physical discomfort (n=67, counts: 600); boring tasks (n=56, counts: 429); and bad weather (n=60, counts: 429).

With regard to stressor severity, the 10 most severe stressors reported in the end-of-week assessment were discrimination or mobbing by another person (n=1, mean 3.0); problem with a pet (n=8, mean 2.4, SD 1.01); conflict or disagreement with close persons (n=51, mean 2.19, SD 0.90); performance situation at work, school, or university (n=42, mean 2.17, SD 1); side effects of medications (n=8, mean 2.16, SD 0.93); high demands or high workload at work, school, or university (n=54, mean 2.15, SD 0.75); bad news (n=22, mean 2.02, SD 1.37); child care problems (n=4, mean 2, SD 0); problem or inconvenience due to house hunting or moving (n=9, mean 2, SD 0.87); and time pressure (n=59, mean 1.98, SD 0.82).

Multimedia Appendix 5 provides an overview of frequency and severity over all three measurement modalities (EMA, end of day, end of week).

### Quantification of Retrospective Bias

Table 2 shows the correlations across subjects between end-of-week, end-of-day, and EMA microstressor assessments in the subjectwise summed microstressor counts, both per week and cumulated for the entire assessment period (week 1 to week 4). With regard to the comparisons per week, all correlation coefficients were high (*r*≥.69), with the highest correlations between the end-of-week and end-of-day comparisons. Regarding the comparison of cumulated data, all correlation coefficients were r≥.83, with the highest correlation again found between end-of-week and end-of-day data.

**Table 2:** Pearson correlations between the end-of-week, end-of-day, and ecological momentary assessments (EMAs) in subjectwise summed microstressor counts (N=70).

Assessment	Week, *r*^a^				
	1	2	3	4	Cumulative (weeks 1-4)
End-of-week vs EMA	.76	.81	.77	.69	.83
End-of-week vs end-of-day	.88	.90	.90	.77	.94
End-of-day vs EMA	.85	.85	.89	.86	.89

^a^*P*<.001 for all correlations.

Table 3 shows the correlations across subjects between end-of-week, end-of-day, and EMA microstressor assessments in the subjectwise averaged severity ratings of all microstressors, both per week and cumulated for the entire assessment period (week 1 to week 4). For the comparisons per week, all correlation coefficients were high (*r*≥.74), with the highest correlations between end-of-week and end-of-day comparisons. Regarding the comparison of cumulated data, all correlation coefficients were *r*≥.85, with the highest correlation again found between end-of-week and end-of-day.

**Table 3:** Pearson correlations between the end-of-week, end-of-day, and ecological momentary assessment microstressor assessments (EMAs) in subjectwise averaged microstressor severity ratings (N=70).

Assessment	Week, *r*^a^				
	1	2	3	4	Cumulative (weeks 1-4)
End-of-week vs EMA	.74	.83	.85	.81	.85
End-of-week vs end-of-day	.84	.90	.91	.90	.95
End-of-day vs EMA	.74	.86	.87	.80	.86

^a^*P*<.001 for all correlations.

Table 4 shows the results of the multilevel modeling analysis for model 1 (total counts of microstressors). The null model showed an ICC of 0.31 for the participants, meaning that 31% of the total variance in the EMA microstressor counts was explained by differences between subjects and 69% by differences within subjects. It also showed an ICC of 0.36, which means that 36% of the total variance in the EMA microstressor counts within persons was explained by differences between weeks and 64% by differences within weeks. The ICC indicated that a large proportion of variance was explained by differences within subjects or within weeks; therefore, we continued with mixed models to account for these within-person/within-week processes. The microstressor counts reported in the end-of-week assessments (weekly, total) predicted the EMA assessments (beta=.03, 95% CI .02-.03, *P*<.001) (see Table 4). That association did not change significantly over the four subsequent weeks (see Table 4: stability assessment). The reported total counts of all microstressors in EMA did not differ significantly between the four weeks (see Table 4: total count of microstressor of each week × week of assessment).

**Table 4:** Multilevel model assessing the association between microstressor counts reported by ecological momentary assessment and end-of-week assessments and potential time-related variations over the course of four subsequent weeks (N=70).

Variable		Beta (SE)	z	*P* value	95% CI
Weekly, total		.03 (.003)	8.67	<.001	.02, .03
**Stability assessment**					
	Week 1	Reference			
	Week 2	−.08 (.14)	−0.62	.53	−.35, .18
	Week 3	−.12 (.14)	−0.86	.39	−.40, .16
	Week 4	−.16 (.16)	−1.00	.32	−.46, .15
**Total counts of microstressors of each week × week of assessment**					
	Week 1	Reference			
	Week 2	−.0006 (.003)	−0.21	.84	−.006, .005
	Week 3	−.00001 (.003)	−0.00	.96	−.006, .006
	Week 4	−.003 (.003)	−0.84	.40	−.009, .004

Table 5 shows the results of the multilevel modeling analysis for model 2 (mean severity of the microstressors). The null model showed an ICC of 0.35 for the participants, meaning that 35% of the total variance in the mean severity of microstressors reported by EMA was explained by differences between subjects and 65% by differences within subjects. It also showed an ICC of 0.42, which means that 42% of the total variance in the EMA assessment within persons was explained by differences between weeks and 58% by differences within weeks. As in the previous section, the ICC indicated that a large proportion of variance was explained by differences within subjects or within weeks. Therefore, we continued with mixed models to account for these within-person/within-week processes. The mean severity of the microstressors reported in the end-of-week assessments (weekly, total) predicted the EMA assessments (beta=.73, 95% CI .59-.88, *P*<.001) (see Table 5). That association did not change significantly over the four subsequent weeks (see Table 5: stability assessment). The reported mean severity of all microstressors in EMA did not differ significantly between the four weeks (see Table 5: mean severity of microstressor of each week × week of assessment).

**Table 5:** Multilevel model assessing the association between the mean severity of microstressors reported by ecological momentary assessment and end-of-week assessments and potential time-related variations over the course of four subsequent weeks (N=70).

Variable		Beta (SE)	z	*P* value	95% CI
Mean severity of all microstressors of each week^a^		.73 (.07)	9.83	<.001	.59, .88
**Stability assessment**					
	Week 1	Reference			
	Week 2	−.19 (.12)	−1.54	.12	−.42, .05
	Week 3	−.15 (.12)	−1.18	.24	−.39, .10
	Week 4	−.14 (.13)	−1.01	.31	−.40, .13
**Mean severity of microstressors of each week × week of assessment**					
	Week 1	Reference			
	Week 2	.07 (.08)	0.93	.36	−.08, .23
	Week 3	.07 (.08)	0.83	.41	−.09, .23
	Week 4	.04 (.09)	0.42	.67	−.13, .21
	Age	−.03 (.01)	−2.58	.01	−.06, −.01

^a^For each selected microstressor, the severity per microstressor was rated using a five-point Likert scale (0, 1, 2, 3, 4; with 0=not at all severe to 4=extremely severe).

## Discussion

### Principal Findings

In this paper, we report the development process and the validation of a retrospective microstressor questionnaire, the Mainz Inventory of Microstressors (MIMIS), which focuses on objective microstressors, includes modern life stressors, and separates stressor occurrence from perceived stressor severity.

In the longitudinal EMA study (study 3), we found high correlations in microstressor counts between the end-of-week, end-of-day, and EMA data (*r*≥.69 for comparisons per week, *r*≥.83 for cumulated data) and high correlations in the mean perceived severity of microstressors between the three measurement methods (*r*≥.74 for comparisons per week, *r*≥.85 for cumulated data). For the reported microstressor counts, the end-of-week questionnaire predicted the EMA assessments sufficiently, and the association did not change significantly over the measurement period of four subsequent weeks. A weaker, although still statistically significant, association was found for microstressor severity. Here again, the association did not change significantly over four subsequent weeks. Our results provide evidence for the ecological validity of the questionnaire.

### Comparison With Existing Scales

Compared with the existing questionnaires [4,11-13,16,17], the MIMIS also includes stressors of modern life due to its recent development. Most of the existing microstressor questionnaires also assess subjective (ie, nonobservable) stressors, except for the Inventory of Small Life Events [12] and the Inventory of College Students’ Recent Life Experiences [17]. However, these questionnaires were developed more than 30 years ago and may not include stressors that result from recent technological or societal developments.

### Strengths and Limitations

A major strength of our study is the use of EMA data for the validation of the questionnaire. To the best of our knowledge, this method has not yet been used in validation studies for microstressor questionnaires. In addition, many of the existing and widely used questionnaires have been developed and validated between 1980 and 1990 [4,11-13]. With developing a new microstressor questionnaire, we were able to include microstressors of modern life. Another strength of our study is the high compliance rate (on average 90%) in the longitudinal EMA study. In addition, the established ecological validity of the MIMIS allows for the quantification of microstressors in a retrospective, low-burden fashion, which does not sacrifice the advantages of EMA in a significant way.

A potential limitation is the length of the questionnaire. However, compared with other microstressor lists, which often include more than 80 items [4,11,17], the MIMIS questionnaire is still a relatively economical assessment method to assess a wide range of microstressors. Another limitation may be the objectivity of the microstressor items. Although we tried to ensure all microstressor items were observable and discretely countable events, one cannot exclude the subjective perception of survey items. The subjective perception may be influenced by individual differences in attention and mood of the participants and consequently influence if someone perceives a particular item as a hassle or not. Another limitation may be that the questionnaire includes microstressors usually occurring in the lives of younger or middle-aged adults. Although some items may apply, we did not specifically take account of microstressors that are most prevalent in older age. Moreover, the samples included in this study were relatively homogeneous for age, education, and employment status. Additional validation studies may be required to test whether the microstressors included in the MIMIS questionnaire are those typically occurring in older age groups and whether there are any differences in samples that are representative of the general population. Two participants reported changes in their behavior due to the assessment in the postmonitoring interview and were excluded from the study. Those participants did not differ from the remaining participants in terms of sociodemographic or psychometric data. There is no reason to assume that the exclusion of those participants introduced bias into the study. In addition, our data do not allow for conclusions on concurrent external validity because data on mental health or other variables related to the effects of microstressor exposure, such as well-being or symptoms of chronic stress, were not assessed at the end of the 28-day period. Future studies should focus on the evaluation of the concurrent external validity by comparing the MIMIS with respective constructs. Another potential limitation may lie in the validation procedure itself, in the way that the repeated EMA assessment could have an effect on the awareness of microstressors that are then reported in the retrospective assessment at the end of the week. Future studies should address that issue by examining potential differences between groups that monitored or did not monitor their microstressors via EMA in the preceding week before completing the MIMIS questionnaire.

### Conclusions and Outlook

In contrast to other microstressor questionnaires that include cognitions, emotions, or consequences of stress, the MIMIS only includes objective stressors. The MIMIS can be applied in basic and applied studies to examine the frequency and perceived severity of a variety of stressors. As applied in this study, it can also be included in the real-life assessment of stressors using mobile technology.

For clinical applications, the MIMIS could serve as a quick and easy-to-administer tool for the assessment of the frequency and the perceived severity of microstressors in the past seven days. In that way, it would provide insight into the current stressor load of the person being investigated. As pointed out elsewhere, the actual stressor load during a period is essential to assess psychological resilience in that period in basic research and intervention studies [22,46].

The aim of this study was to develop the questionnaire and assess the ecological validity of the MIMIS by quantifying the potential retrospective bias. Future studies should focus on the external validation of the MIMIS by, for example, comparing the subjective severity of microstressors reported in MIMIS with biological markers for stress response, such as cortisol levels [14]. In that way, it could be investigated whether microstressors that are subjectively rated as more severe also lead to higher stress responses, as would be expected.

This study provides evidence for the ecological validity of the MIMIS. In future studies, the questionnaire should be tested on other age groups, such as older adults or teenagers.

## Appendix

Multimedia Appendix 1 Overview of self-report scales for the assessment of microstressor considered for the development of MIMIS.

Multimedia Appendix 2 Item generation.

Multimedia Appendix 3 Checklist for Reporting Results of Internet E-Surveys (CHERRIES).

Multimedia Appendix 4 Mainz Inventory of Microstressors (MIMIS).

Multimedia Appendix 5 Counts and average severity of each microstressor.

## Abbreviations

EMA: ecological momentary assessment
ICC: intraclass correlation
MIMIS: Mainz Inventory of Microstressors
