# Correction: Assessment of Microstressors in Adults: Questionnaire Development and Ecological Validation of the Mainz Inventory of Microstressors

**DOI:** 10.2196/18626

**Published:** 2020-05-04

**Authors:** Andrea Chmitorz, Karolina Kurth, Lara K Mey, Mario Wenzel, Klaus Lieb, Oliver Tüscher, Thomas Kubiak, Raffael Kalisch

**Affiliations:** 1 Department of Psychiatry and Psychotherapy University Medical Center Mainz Mainz Germany; 2 Faculty of Social Work, Health Care and Nursing Sciences Esslingen University of Applied Sciences Esslingen Germany; 3 Health Psychology Institute for Psychology Johannes Gutenberg University Mainz Germany; 4 Leibniz Institute for Resilience Research Mainz Germany; 5 Neuroimaging Center University Medical Center Mainz Germany

The authors of “Assessment of Microstressors in Adults: Questionnaire Development and Ecological Validation of the Mainz Inventory of Microstressors” (JMIR Ment Health 2020;7(2):e14566) noticed that the equal contribution footnote was missing from the author list.

This has been amended to indicate that authors Oliver Tüscher, Thomas Kubiak, and Raffael Kalisch all contributed equally.

The correction will appear in the online version of the paper on the JMIR website on May 4, together with the publication of this correction notice. Because this was made after submission to PubMed, PubMed Central, and other full-text repositories, the corrected article has also been resubmitted to those repositories.

