# Supplementary material for: Assessment of Microstressors in Adults: Questionnaire Development and Ecological Validation of the Mainz Inventory of Microstressors
Source: JMIR Ment Health. 2020 Feb 24;7(2):e14566. doi: 10.2196/14566 (PMC7063526; doi:10.2196/14566)
Supplement: Multimedia Appendix 1 [file mental_v7i2e14566_app1.docx]

**Appendix 1: Overview of self-report scales for the assessment of microstressor considered for the development of MIMIS**

| **Name and reference:** | **Aim of measurement:** | **Number of items:** | **Scale:** | **Assessment period:** |
| --- | --- | --- | --- | --- |
| Hassles and Uplifts Scales [11] | Occurrence and perceived severity of hassles.  Occurrence of uplifts. | 117 hassles  135 uplifts | Four-point Likert scale for the assessment of hassles (0 = ‘did not occur’, 1 = ‘somewhat severe’, 2 = ‘moderately severe’, 3 = ‘extremely severe’) and uplifts (0 = ‘did not occur’, 1 = ‘somewhat often’, 2 = ‘moderately often’, 3 = ‘extremely often’). | Past 4 weeks |
| Inventory of Small Life Events’  [12] | Readjustment, controllability, desirability and locus of causation of observable events | 178 desirable and undesirable observable events | Readjustment (numeric value), controllability (four-point Likert scale), desirability (from -5 = ‘extremely undesirable’ to 5 = ‘extremely desirable’) and locus of causation (1 = ‘completely due to the person’ to 5 = ‘completely due to others/ circumstances’) for each event. | Past 4 weeks |
| Daily Stress Inventory’  [13] | Assessment of potentially stressful events, rating of the amount of stress caused per event. | 58 items | Occurrence of potentially stressful event (yes/no)  Rating of the amount of stress caused per event (X = ‘did not occur’, 1 = ‘occurred but was not stressful’, 2 = ‘caused very little stress’, 3 = ‘caused a little stress’, 4 = ‘caused some stress’, 5 = ‘caused much stress’, 6 = ‘caused very much stress’, 7 = ‘caused me to panic’). | Past 24 hours |
| ‘The Weekly Hassle Scale’  [14] | Retrospective assessment of microstressors | 30 items | Using a five-point Likert scale, participants are asked how often each hassle occurred during the past seven days (0 = ‘did not occur’ to 4 = ‘occurred always’) and how stressful it has been for them (0 = ‘not at all stressful’ to 4 = ‘extremely stressful’). | Past 7 days |
| ‘Adolescent Stress Questionnaire’  [15] | Assess adolescent stress | 58 items | Each item was rated on a five-point Likert scale (1 = ‘not at all stressful (or is irrelevant to me)’, 2 = ‘a little stressful’, 3 = ‘moderately stressful’, 4 = ‘quite stressful’, 5 = ‘very stressful’). | Past 12 months |
| ‘Everyday Stressor Index’  [16] | Assessment of microstressors occurring in everyday life of Turkish or German mothers with young children | 19 items with items taken from HASSUP and additional items that are relevant for the target group | Stressors are rated on a four-point Likert scale (1 = ‘does not affect me at all’, 2 = ‘does affect me a little bit’, 3 = ‘does affect me a bit more’, 4 = ‘does affect me a lot’). | Not stated |
| ‘Inventory of College Students' Recent Life Experiences’  [17] | Assessment of objective stressors in students | 85 items | Objective stressor items from existing scales and additional items. The perceived impact of the stressor is assessed separately by the reported extent to which each stressor was experienced (1 = ‘not at all part of my life’, 2 = ‘only slightly part of my life’, 3 = distinctly part of my life, 4 = ‘very much part of my life’). | Past 4 weeks |
