# Supplementary material for: Assessment of Microstressors in Adults: Questionnaire Development and Ecological Validation of the Mainz Inventory of Microstressors
Source: JMIR Ment Health. 2020 Feb 24;7(2):e14566. doi: 10.2196/14566 (PMC7063526; doi:10.2196/14566)
Supplement: Multimedia Appendix 2 [file mental_v7i2e14566_app2.docx]

**Appendix 2: Item generation**

In a first step, we examined the literature on microstressors or daily hassles scales to identify hassles included in previous scales which were developed for adolescents and adults [11, 15, 16]. As a result, 49 items were drawn from scales in English language and were first translated in the German language and then back translated in English. In a next step, we conducted an expert group, consisting of experts from the fields of health psychology, stress research, psychiatry, and clinical psychology. The members of the expert group were asked to determine the kind of stressors associated with everyday life. The results were used to (1) confirm the relevance and (2) review the wording of the items already identified from the literature, and (3) identify additional items reflecting the relevant stressors of modern life. Additional 26 items were added, including stressors related to aspects of modern life, such as digitalization or urbanization.

We then conducted a feasibility study with three undergraduate students (N = 2 women, N = 1 man, age range = 22-26 years) and four PhD students (N = 3 women, N = 1 man, age range = 23-35 years). After completing the questionnaire, semi-structured interviews were conducted with each participant to detect critical items that were ambiguous or difficult to understand and to identify additional items that could be added to the list. The items were then independently reviewed by the authors and modified after we reached consensus by discussion. This list of items and the suggested scale were then shown to experts in the field of health psychology and psychometrics. This resulted in 23 items being revised, and eight items being removed. The first version of the questionnaire included 67 microstressor items. Participants were asked to provide information about microstressors occurring during the past seven days (‘on approximately how many days did the situation occur?’). In addition, participants could add up to five additional microstressors that were relevant to them but not yet covered in the questionnaire. In order to obtain additional information about the individual impact of each stressor, the questionnaire includes a five-point Likert scale (0, 1, 2, 3, 4; with 0 = ‘not at all severe’ to 4 = ‘extremely severe’) after each item, asking for the perceived severity of the stressor (‘on average, to what extent did you find the situations straining?’).
