# Supplementary material for: Assessment of Microstressors in Adults: Questionnaire Development and Ecological Validation of the Mainz Inventory of Microstressors
Source: JMIR Ment Health. 2020 Feb 24;7(2):e14566. doi: 10.2196/14566 (PMC7063526; doi:10.2196/14566)
Supplement: Multimedia Appendix 3 [file mental_v7i2e14566_app3.docx]

**Appendix 3: Checklist for Reporting Results of Internet E-Surveys (CHERRIES)**

| **Item category** | **Checklist item** |  |
| --- | --- | --- |
| Design | Describe survey design | Study 1: Cross-sectional study (N=108).  Study 2 and 3: Longitudinal studies (N=10 and N=70) using Ecological Momentary Assessment (EMA) to compare stressor data, collected five times per day for 30 days, with retrospective reports (end-of-day, -week).  All participants were recruited via flyers at the Johannes Gutenberg University, Mainz, Germany. |
| IRB (Institutional Review Board) approval and informed consent process | IRB approval | The study protocols were approved by the ethics committee at the Rhineland-Palatinate state chamber of physicians (837.085.13 [8770-F] and 837.183.16 [10502]) |
|  | Informed consent | For all studies, potential participants were invited for an initial briefing session, where information on the purpose of the survey, the length of time, the investigator and data storage was provided and written consent was obtained. |
|  | Data protection | Online-surveys using ‘soscisurvey’:  Soscisurvey uses continuous SSL encryption (HTTPS), a secure SSL configuration (Qualys SSL Labs) for retrieving and transmitting the data, the use of proven software components (operating system [Ubuntu Linux], server application [nginx], database [MySQL], end encryption of backup copies [GPG]), security updates for the software packages several times a day and a server that is located in the certified and secured data center of the provider M-net Telekommunikations GmbH to protect against unauthorized data access (soscisurvey, 2019).  Collection of EMA-data using ‘Movisens’:  Movisens uses a 256 Bit encrypted data transfer on all data channels (between smartphone and server respectively server and browser of the researcher), 256 Bit encrypted data storage on the smartphone. Decryption of the data will happen only on the server. If a smartphone is lost by the participant, the data collected cannot be decrypted by a third person. The physical servers are hosted by the certified and secured data center of the provider TelemaxX Telekommunikation GmbH in Karlsruhe, Germany. |
| Development and pre-testing | Development and testing | - Questionnaire development: - Literature on microstressor and daily hassles scales was examined to identify hassles included in previous scales which were developed for adolescents and adults, resulting in 49 items drawn from those scales. - Translation and back translation - Expert group to confirm the relevance and review the wording of the items already identified from the literature, and identify additional items reflecting the relevant stressors of modern life. Additional 26 items were added. - Feasibility study (N= 5) including semi-structured interviews to detect and revise critical items. - Cross-sectional study (study 1) to evaluate the 67-item version of the questionnaire - Small-scale EMA feasibility study (study 2) to test the feasibility of a smartphone-based EMA assessment of the questionnaire. |
| Recruitment process and description of the sample having access to the questionnaire | Open survey versus closed survey | Closed surveys. All surveys were password-protected and could only be accessed by invitation. |
|  | Contact mode | Initial contact was made via phone or e-mail. The potential participants were then invited to the initial briefing session (face-to-face). After written consent was obtained and inclusion criteria were met each participant was registered received a unique identification code and log-in data for the soscisurvey database. |
|  | Advertising the survey | Participants were recruited via flyers that were distributed on and posters which were displayed at the University campus. |
| Survey administration | Web/E-mail | The study participants logged in at the soscisurvey webpage using their personalized access data. The questionnaires were then completed by the participants and the data stored in a database. |
|  | Context | Not applicable, the survey was not posted on an external web site. |
|  | Mandatory/voluntary | Not applicable, the participants visited the soscisurvey platform for the purpose of entering the data. |
|  | Incentives | Study 1: all participants were entered into a prize draw for book vouchers.  Study 2 and study 3: The participants received a monetary compensation at end of the study. Here we applied a scoring system, in order to increase the motivation to participate in the study and provide complete data sets. The score accounted for the number of complete datasets provided in the EMA assessments and the online questionnaires.  The scoring system consisted of a basic compensation and an additional bonus:  Basic compensation: For a complete dataset (100%) participants received a basic compensation of 100 Euros. The amount was decreasing depending on the percentage completed (e. g., 98 % resulted in 98 Euros).  Bonus:  a) in the daily assessment, for answering at least four EMA-signals and completing the end-of-day assessment, participants received additional 2 Euros per day (which translates to a total of 28 days x 2 Euros = 56 Euros)  b) at the end of the assessment period, if at least 28 EMA signals and all seven end-of-day assessments were completed, participants received additional 5 Euros per week (translating to four weeks x 5 Euros = 20 Euros). Participants received up to 176 Euros in study 3 and the proportionate amount for one week in study 2. |
|  | Time/Date | Study 1: October 2014 to January 2016  Study 2: June 2016  Study 3: September 2016 and March 2017 |
|  | Randomization of items or questionnaire | The items or questionnaires were not randomized. |
|  | Adaptive questioning | Adaptive questioning was not applied. |
|  | Number of Items | Weekly and daily assessment using ‘soscisurvey’ (study 1, 2, and 3): ten to twelve items per page  EMA-assessment using ‘Movisens’: a list of 59 daily hassles was provided. If a hassle was selected, the severity for that hassle was assessed on a new page. |
|  | Number of screens (pages) | Weekly and daily assessment using ‘soscisurvey’ (used in study 1, 2, and 3):  Study 1 and 2: 16 pages for the 67-item questionnaire  Study 3: 14 pages for the final 58-item questionnaire  EMA-assessment using ‘Movisens’:   - List of daily hassles: one page - Severity rating: one page per selected hassle |
|  | Completeness check | All items were provided with a non-response option (“not applicable”, “I rather not say”), forced response was applied to obtain complete datasets. |
|  | Review step | Respondents were able to change their answers through a back button. After completing the questionnaire and submitting the data, data could not be changed by the participants. |
| Response rates | Unique site visitor | Not applicable. The participants received a unique identification code and accessed the online survey by invitation. |
|  | View rate (Ratio unique site visitors/unique survey visitors) | Not applicable. The participants received a unique identification code and accessed the online survey by invitation. |
|  | Participation rate (Ratio unique survey page visitors/agreed to participate) | Not applicable. The participants received a unique identification code and accessed the online survey by invitation. |
|  | Completion rate (Ratio agreed to participate/finished survey) | There were no incomplete surveys. Two participants dropped out during the study, but completed all surveys during the time they participated in the study. |
| Preventing multiple entries from the same individual | Cookies used | Not applicable. The participants received a unique identification code. There were no duplicate entries. Duplicate entries would have been deleted. |
|  | IP check | Not applicable. The participants received a unique identification code. There were no duplicate entries. Duplicate entries would have been deleted. |
|  | Log file analysis | Not applicable. The participants received a unique identification code. There were no duplicate entries. Duplicate entries would have been deleted. |
|  | Registration | The participants received a unique identification code and accessed the online survey by invitation via email. |
| Analysis | Handling of incomplete questionnaires | Not applicable. Forced response including a non-response option (“not applicable”, “I rather not say”) was used to prevent missing data. |
|  | Questionnaires submitted with an atypical timestamp | Not applicable. No timeframe was used as cut-off point for the online-questionnaire. |
|  | Statistical correction | Methods to adjust for potential non-representativeness of the sample (eg, weighting or propensity scores) were not applied. |

Adopted from:

Eysenbach G. Improving the quality of web surveys: the checklist for reporting results of internet e-surveys (cherries). J Med Internet Res 2004;6(3):e34.
