# Supplementary material for: Assessment of Microstressors in Adults: Questionnaire Development and Ecological Validation of the Mainz Inventory of Microstressors
Source: JMIR Ment Health. 2020 Feb 24;7(2):e14566. doi: 10.2196/14566 (PMC7063526; doi:10.2196/14566)
Supplement: Multimedia Appendix 4 [file mental_v7i2e14566_app4.docx]

**Appendix 4: Mainz Inventory of Microstressors (MIMIS)**

**Mainz Inventory of Microstressors (MIMIS) – German version**

Nun möchten wir Sie über mögliche **Ärgernisse oder Unannehmlichkeiten** befragen, die im **täglichen Leben** auftreten können.

Die nachfolgende Liste enthält eine Sammlung an möglichen Situationen. Bitte denken Sie jetzt nur an die **vergangenen 7 Tage bis einschließlich heute**.
Bitte lesen Sie die Liste der Reihe nach durch und bewerten Sie:

- An **wie vielen Tagen** die jeweilige Situation schätzungsweise auftrat (bitte wählen Sie ‚trat nicht auf‘', falls sie die jeweilige Situation nicht erlebt haben),
- Wie **belastend** Sie die jeweilige Situation durchschnittlich erlebt haben (‚gar nicht belastend‘ bis ‚sehr belastend‘).

Bitte denken Sie jetzt nur an die **vergangenen 7 Tage bis einschließlich heute**.

|  | **An wie vielen Tagen trat die Situation schätzungsweise auf?** | | | | | | | | | | | | |  | | **Wie belastend haben  Sie die Situation  durchschnittlich erlebt?** |  |
| --- | --- | --- | --- | --- | --- | --- | --- | --- | --- | --- | --- | --- | --- | --- | --- | --- | --- |
|  | **1** | | **2** | | **3** | | **4** | | **5** | | **6** | | **7** |  | | **gar**  **nicht sehr** | **trat  nicht auf** |
| 1. Verlieren oder Verlegen von Gegenständen | ⭘ | | ⭘ | | ⭘ | | ⭘ | | ⭘ | | ⭘ | | ⭘ |  | | ⭘ ⭘ ⭘ ⭘ ⭘ | 🞎 |
| 1. Negatives Ereignis in den Medien | ⭘ | | ⭘ | | ⭘ | | ⭘ | | ⭘ | | ⭘ | | ⭘ |  | | ⭘ ⭘ ⭘ ⭘ ⭘ | 🞎 |
| 1. Negatives politisches Ereignis | ⭘ | | ⭘ | | ⭘ | | ⭘ | | ⭘ | | ⭘ | | ⭘ |  | | ⭘ ⭘ ⭘ ⭘ ⭘ | 🞎 |
| 1. Gesellschaftliche Verpflichtung | ⭘ | | ⭘ | | ⭘ | | ⭘ | | ⭘ | | ⭘ | | ⭘ |  | | ⭘ ⭘ ⭘ ⭘ ⭘ | 🞎 |
| 1. Unterbrechung bei einer Tätigkeit (z. B. in der Arbeit oder bei einer Freizeitbeschäftigung) | ⭘ | | ⭘ | | ⭘ | | ⭘ | | ⭘ | | ⭘ | | ⭘ |  | | ⭘ ⭘ ⭘ ⭘ ⭘ | 🞎 |
| 1. Wartezeit oder Verzögerung (z. B. bei Bus oder Bahn, Warten auf eine Person) | ⭘ | | ⭘ | | ⭘ | | ⭘ | | ⭘ | | ⭘ | | ⭘ |  | | ⭘ ⭘ ⭘ ⭘ ⭘ | 🞎 |
| 1. Flüchtigkeitsfehler oder Fehler wegen mangelnder Aufmerksamkeit | ⭘ | | ⭘ | | ⭘ | | ⭘ | | ⭘ | | ⭘ | | ⭘ |  | | ⭘ ⭘ ⭘ ⭘ ⭘ | 🞎 |
| 1. Gerede oder Lästereien von anderen Leuten (einschließlich in sozialen Medien) | ⭘ | | ⭘ | | ⭘ | | ⭘ | | ⭘ | | ⭘ | | ⭘ |  | | ⭘ ⭘ ⭘ ⭘ ⭘ | 🞎 |
| 1. Diskriminierung oder Mobbing durch eine andere Person (einschließlich in sozialen Medien) | ⭘ | | ⭘ | | ⭘ | | ⭘ | | ⭘ | | ⭘ | | ⭘ |  | | ⭘ ⭘ ⭘ ⭘ ⭘ | 🞎 |
| 1. Alpträume | ⭘ | | ⭘ | | ⭘ | | ⭘ | | ⭘ | | ⭘ | | ⭘ |  | | ⭘ ⭘ ⭘ ⭘ ⭘ | 🞎 |
| 1. Anfahrt/Pendeln zur Arbeit/Ausbildungsstelle/ Schule/Hochschule | ⭘ | | ⭘ | | ⭘ | | ⭘ | | ⭘ | | ⭘ | | ⭘ |  | | ⭘ ⭘ ⭘ ⭘ ⭘ | 🞎 |
| 1. Kleinerer Gesetzesverstoß (z. B. Bußgeld wegen Ordnungswidrigkeit) | ⭘ | | ⭘ | | ⭘ | | ⭘ | | ⭘ | | ⭘ | | ⭘ |  | | ⭘ ⭘ ⭘ ⭘ ⭘ | 🞎 |
| 1. Unannehmlichkeit mit Behörde, Amt oder anderer Institution (z. B. Finanzamt, Bank, Firma) | ⭘ | | ⭘ | | ⭘ | | ⭘ | | ⭘ | | ⭘ | | ⭘ |  | | ⭘ ⭘ ⭘ ⭘ ⭘ | 🞎 |
| 1. Konflikt oder Meinungsverschiedenheit am Arbeitsplatz (z. B. mit Vorgesetzten oder Arbeitskollegen) | ⭘ | | ⭘ | | ⭘ | | ⭘ | | ⭘ | | ⭘ | | ⭘ |  | | ⭘ ⭘ ⭘ ⭘ ⭘ | 🞎 |
| 1. Konflikt oder Meinungsverschiedenheit mit nahestehenden Personen (z. B. Eltern, Geschwister, Partnerin/Partner) | ⭘ | | ⭘ | | ⭘ | | ⭘ | | ⭘ | | ⭘ | | ⭘ |  | | ⭘ ⭘ ⭘ ⭘ ⭘ | 🞎 |
| 1. Konflikt oder Meinungsverschiedenheit zwischen nahestehenden Personen (z. B. zwischen den Eltern, zwischen Freunden/ Freundinnen) | ⭘ | | ⭘ | | ⭘ | | ⭘ | | ⭘ | | ⭘ | | ⭘ |  | | ⭘ ⭘ ⭘ ⭘ ⭘ | 🞎 |
| 1. Konflikt oder Meinungsverschiedenheit mit anderen nicht-nahestehenden Personen (z. B. Busfahrer, Nachbar) | ⭘ | | ⭘ | | ⭘ | | ⭘ | | ⭘ | | ⭘ | | ⭘ |  | | ⭘ ⭘ ⭘ ⭘ ⭘ | 🞎 |
|  | | **An wie vielen Tagen trat die Situation schätzungsweise auf?** | | | | | | | | | | | |  | **Wie belastend haben  Sie die Situation  durchschnittlich erlebt?** | |  |
|  |  | **1** | | **2** | | **3** | | **4** | | **5** | | **6** | **7** |  | **gar**  **nicht sehr** | | **trat  nicht auf** |
| 1. Konflikt oder Meinungsverschiedenheit mit Ihrem Kind/ Ihren Kindern | | ⭘ | | ⭘ | | ⭘ | | ⭘ | | ⭘ | | ⭘ | ⭘ |  | ⭘ ⭘ ⭘ ⭘ ⭘ | | 🞎 |
| 1. Problem mit der Kinderbetreuung | | ⭘ | | ⭘ | | ⭘ | | ⭘ | | ⭘ | | ⭘ | ⭘ |  | ⭘ ⭘ ⭘ ⭘ ⭘ | | 🞎 |
| 1. Besorgung oder Fahrdienst für andere (z. B. Medikament für Familienmitglied besorgen) | | ⭘ | | ⭘ | | ⭘ | | ⭘ | | ⭘ | | ⭘ | ⭘ |  | ⭘ ⭘ ⭘ ⭘ ⭘ | | 🞎 |
| 1. Problem oder Unannehmlichkeit dadurch, dass Ihre Freunde oder Verwandten zu weit weg wohnen | | ⭘ | | ⭘ | | ⭘ | | ⭘ | | ⭘ | | ⭘ | ⭘ |  | ⭘ ⭘ ⭘ ⭘ ⭘ | | 🞎 |
| 1. Problem durch fehlende Unterstützung oder Hilfe durch andere | | ⭘ | | ⭘ | | ⭘ | | ⭘ | | ⭘ | | ⭘ | ⭘ |  | ⭘ ⭘ ⭘ ⭘ ⭘ | | 🞎 |
| 1. Problem mit Ihrem Haustier (z. B. Krankheit, unerwünschtes Verhalten) | | ⭘ | | ⭘ | | ⭘ | | ⭘ | | ⭘ | | ⭘ | ⭘ |  | ⭘ ⭘ ⭘ ⭘ ⭘ | | 🞎 |
| 1. Beeinträchtigung durch unsichere Umgebung (z. B. unsicheres Wohnumfeld) | | ⭘ | | ⭘ | | ⭘ | | ⭘ | | ⭘ | | ⭘ | ⭘ |  | ⭘ ⭘ ⭘ ⭘ ⭘ | | 🞎 |
| 1. Beeinträchtigung durch Dreck, Verschmutzung oder Gestank (z. B. in der Wohngegend oder Wohnung) | | ⭘ | | ⭘ | | ⭘ | | ⭘ | | ⭘ | | ⭘ | ⭘ |  | ⭘ ⭘ ⭘ ⭘ ⭘ | | 🞎 |
| 1. Problem dadurch, dass nicht genügend Geld zur Verfügung steht (z. B. für Grundversorgung, Notfälle oder besondere Wünsche) | | ⭘ | | ⭘ | | ⭘ | | ⭘ | | ⭘ | | ⭘ | ⭘ |  | ⭘ ⭘ ⭘ ⭘ ⭘ | | 🞎 |
| 1. Andere schulden Ihnen Geld | | ⭘ | | ⭘ | | ⭘ | | ⭘ | | ⭘ | | ⭘ | ⭘ |  | ⭘ ⭘ ⭘ ⭘ ⭘ | | 🞎 |
| 1. Sie schulden anderen Geld | | ⭘ | | ⭘ | | ⭘ | | ⭘ | | ⭘ | | ⭘ | ⭘ |  | ⭘ ⭘ ⭘ ⭘ ⭘ | | 🞎 |
| 1. Hohe oder unerwartete finanzielle Belastung (z. B. Kauf teurer Produkte oder Gegenstände, Kosten für Reparatur des Autos) | | ⭘ | | ⭘ | | ⭘ | | ⭘ | | ⭘ | | ⭘ | ⭘ |  | ⭘ ⭘ ⭘ ⭘ ⭘ | | 🞎 |
| 1. Finanzielle Angelegenheit (z. B. Rechnungen bezahlen, Beschäftigung mit finanzieller Vorsorge im Alter) | | ⭘ | | ⭘ | | ⭘ | | ⭘ | | ⭘ | | ⭘ | ⭘ |  | ⭘ ⭘ ⭘ ⭘ ⭘ | | 🞎 |
| 1. Unerwarteter oder unerwünschter Besuch | | ⭘ | | ⭘ | | ⭘ | | ⭘ | | ⭘ | | ⭘ | ⭘ |  | ⭘ ⭘ ⭘ ⭘ ⭘ | | 🞎 |
| 1. Nebenwirkung von Medikamenten | | ⭘ | | ⭘ | | ⭘ | | ⭘ | | ⭘ | | ⭘ | ⭘ |  | ⭘ ⭘ ⭘ ⭘ ⭘ | | 🞎 |
| 1. Eigene körperliche Beschwerde (z. B. leichtere Erkrankung oder Schmerzen) | | ⭘ | | ⭘ | | ⭘ | | ⭘ | | ⭘ | | ⭘ | ⭘ |  | ⭘ ⭘ ⭘ ⭘ ⭘ | | 🞎 |
| 1. Körperliche Beschwerde (z. B. leichtere Erkrankung oder Schmerzen) bei einer nahestehenden Person | | ⭘ | | ⭘ | | ⭘ | | ⭘ | | ⭘ | | ⭘ | ⭘ |  | ⭘ ⭘ ⭘ ⭘ ⭘ | | 🞎 |

|  | **An wie vielen Tagen trat die Situation schätzungsweise auf?** | | | | | | |  | **Wie belastend haben  Sie die Situation  durchschnittlich erlebt?** |  |
| --- | --- | --- | --- | --- | --- | --- | --- | --- | --- | --- |
|  | **1** | **2** | **3** | **4** | **5** | **6** | **7** |  | **gar**  **nicht sehr** | **trat  nicht auf** |
| 1. Schlafmangel oder Schlafprobleme | ⭘ | ⭘ | ⭘ | ⭘ | ⭘ | ⭘ | ⭘ |  | ⭘ ⭘ ⭘ ⭘ ⭘ | 🞎 |
| 1. Arztbesuch | ⭘ | ⭘ | ⭘ | ⭘ | ⭘ | ⭘ | ⭘ |  | ⭘ ⭘ ⭘ ⭘ ⭘ | 🞎 |
| 1. Zuhause anfallende Büro- oder Schreibarbeit (z. B. Formulare ausfüllen) | ⭘ | ⭘ | ⭘ | ⭘ | ⭘ | ⭘ | ⭘ |  | ⭘ ⭘ ⭘ ⭘ ⭘ | 🞎 |
| 1. Haushaltsführung (z. B. Kochen, Putzen oder Einkaufen) | ⭘ | ⭘ | ⭘ | ⭘ | ⭘ | ⭘ | ⭘ |  | ⭘ ⭘ ⭘ ⭘ ⭘ | 🞎 |
| 1. Vornehmen einer kleineren Reparatur (z. B. im eigenen Haus) | ⭘ | ⭘ | ⭘ | ⭘ | ⭘ | ⭘ | ⭘ |  | ⭘ ⭘ ⭘ ⭘ ⭘ | 🞎 |
| 1. Problem mit einem technischen Gerät (z. B. Computer, Haushaltsgerät, Elektrogerät) | ⭘ | ⭘ | ⭘ | ⭘ | ⭘ | ⭘ | ⭘ |  | ⭘ ⭘ ⭘ ⭘ ⭘ | 🞎 |
| 1. Wartung oder Instandhaltung eines Gegenstands (z. B. des Autos) | ⭘ | ⭘ | ⭘ | ⭘ | ⭘ | ⭘ | ⭘ |  | ⭘ ⭘ ⭘ ⭘ ⭘ | 🞎 |
| 1. Unangenehmes oder schlechtes Wetter (z. B. Regen, Hitze, Kälte) | ⭘ | ⭘ | ⭘ | ⭘ | ⭘ | ⭘ | ⭘ |  | ⭘ ⭘ ⭘ ⭘ ⭘ | 🞎 |
| 1. Störendes Verhalten oder Fehlverhalten anderer (z. B. rücksichtslose Raucher, störende Nachbarn) | ⭘ | ⭘ | ⭘ | ⭘ | ⭘ | ⭘ | ⭘ |  | ⭘ ⭘ ⭘ ⭘ ⭘ | 🞎 |
| 1. Schlechtes Essen (z. B. in der Mensa oder Kantine) | ⭘ | ⭘ | ⭘ | ⭘ | ⭘ | ⭘ | ⭘ |  | ⭘ ⭘ ⭘ ⭘ ⭘ | 🞎 |
| 1. Lärm (z. B. Straßenlärm, Fluglärm) | ⭘ | ⭘ | ⭘ | ⭘ | ⭘ | ⭘ | ⭘ |  | ⭘ ⭘ ⭘ ⭘ ⭘ | 🞎 |
| 1. Verkehrsstau | ⭘ | ⭘ | ⭘ | ⭘ | ⭘ | ⭘ | ⭘ |  | ⭘ ⭘ ⭘ ⭘ ⭘ | 🞎 |
| 1. Suche nach einem Parkplatz | ⭘ | ⭘ | ⭘ | ⭘ | ⭘ | ⭘ | ⭘ |  | ⭘ ⭘ ⭘ ⭘ ⭘ | 🞎 |
| 1. Problem mit einem Kommunikationsmittel (z. B. Internet, Telefon) | ⭘ | ⭘ | ⭘ | ⭘ | ⭘ | ⭘ | ⭘ |  | ⭘ ⭘ ⭘ ⭘ ⭘ | 🞎 |
| 1. Leistungssituation in der Arbeit/Schule/Studium (z. B. Prüfung) | ⭘ | ⭘ | ⭘ | ⭘ | ⭘ | ⭘ | ⭘ |  | ⭘ ⭘ ⭘ ⭘ ⭘ | 🞎 |
| 1. Hohe Leistungsanforderung oder hohes Arbeitspensum in der Arbeit/Schule/Studium | ⭘ | ⭘ | ⭘ | ⭘ | ⭘ | ⭘ | ⭘ |  | ⭘ ⭘ ⭘ ⭘ ⭘ | 🞎 |
| 1. Langweilige Tätigkeit (z. B. in der Arbeit oder im Studium) | ⭘ | ⭘ | ⭘ | ⭘ | ⭘ | ⭘ | ⭘ |  | ⭘ ⭘ ⭘ ⭘ ⭘ | 🞎 |

|  | **An wie vielen Tagen trat die Situation schätzungsweise auf?** | | | | | | |  | **Wie belastend haben  Sie die Situation  durchschnittlich erlebt?** |  |
| --- | --- | --- | --- | --- | --- | --- | --- | --- | --- | --- |
|  | **1** | **2** | **3** | **4** | **5** | **6** | **7** |  | **gar**  **nicht sehr** | **trat  nicht auf** |
| 1. Besprechung (z. B. in der Arbeit, im Studium, im Verein) | ⭘ | ⭘ | ⭘ | ⭘ | ⭘ | ⭘ | ⭘ |  | ⭘ ⭘ ⭘ ⭘ ⭘ | 🞎 |
| 1. Ungeregelte oder zu lange Arbeitszeiten | ⭘ | ⭘ | ⭘ | ⭘ | ⭘ | ⭘ | ⭘ |  | ⭘ ⭘ ⭘ ⭘ ⭘ | 🞎 |
| 1. Problem mit der Planung oder Vereinbarung von Terminen | ⭘ | ⭘ | ⭘ | ⭘ | ⭘ | ⭘ | ⭘ |  | ⭘ ⭘ ⭘ ⭘ ⭘ | 🞎 |
| 1. Zeitdruck | ⭘ | ⭘ | ⭘ | ⭘ | ⭘ | ⭘ | ⭘ |  | ⭘ ⭘ ⭘ ⭘ ⭘ | 🞎 |
| 1. Schlechte Neuigkeit (z. B. Absage auf Bewerbung, Mitteilung über schlechtes Prüfungsergebnis) | ⭘ | ⭘ | ⭘ | ⭘ | ⭘ | ⭘ | ⭘ |  | ⭘ ⭘ ⭘ ⭘ ⭘ | 🞎 |
| 1. Problem oder Unannehmlichkeit durch Suche nach einem Ausbildungs-/Studien-/ oder Arbeitsplatz | ⭘ | ⭘ | ⭘ | ⭘ | ⭘ | ⭘ | ⭘ |  | ⭘ ⭘ ⭘ ⭘ ⭘ | 🞎 |
| 1. Problem oder Unannehmlichkeit durch Wohnungssuche oder Umzug | ⭘ | ⭘ | ⭘ | ⭘ | ⭘ | ⭘ | ⭘ |  | ⭘ ⭘ ⭘ ⭘ ⭘ | 🞎 |

**Mainz Inventory of Microstressors (MIMIS) – English version**

We would like to ask you about annoyances and hassles as may occur in daily life.

The list below contains possible situations. Please only consider **the last 7 days including today.** Please read each item on the list and rate:

- On **approximately how many days** the mentioned situation occurred (please choose ‘did not occur’ if you did not experience the situation)
- To **what extent** the situation caused you mental **strain** (from ‘not at all’ to ‘very straining’)

Please think about **the last 7 days including today.**

|  | **On approximately how many days did the situation occur?** | | | | | | | | | | | | |  | | **To what extent did you find the situation mentally straining, on average?** |  |
| --- | --- | --- | --- | --- | --- | --- | --- | --- | --- | --- | --- | --- | --- | --- | --- | --- | --- |
|  | **1** | | **2** | | **3** | | **4** | | **5** | | **6** | | **7** |  | | **Not at all Very** | **Did not occur** |
| 1. Losing or displacing objects | ⭘ | | ⭘ | | ⭘ | | ⭘ | | ⭘ | | ⭘ | | ⭘ |  | | ⭘ ⭘ ⭘ ⭘ ⭘ | 🞎 |
| 1. Negative event in the media | ⭘ | | ⭘ | | ⭘ | | ⭘ | | ⭘ | | ⭘ | | ⭘ |  | | ⭘ ⭘ ⭘ ⭘ ⭘ | 🞎 |
| 1. Negative political event | ⭘ | | ⭘ | | ⭘ | | ⭘ | | ⭘ | | ⭘ | | ⭘ |  | | ⭘ ⭘ ⭘ ⭘ ⭘ | 🞎 |
| 1. Social obligation | ⭘ | | ⭘ | | ⭘ | | ⭘ | | ⭘ | | ⭘ | | ⭘ |  | | ⭘ ⭘ ⭘ ⭘ ⭘ | 🞎 |
| 1. Interruption during an activity (eg, at work or during leisure activities) | ⭘ | | ⭘ | | ⭘ | | ⭘ | | ⭘ | | ⭘ | | ⭘ |  | | ⭘ ⭘ ⭘ ⭘ ⭘ | 🞎 |
| 1. Waiting time or delay (eg, waiting for a person; bus or train delay) | ⭘ | | ⭘ | | ⭘ | | ⭘ | | ⭘ | | ⭘ | | ⭘ |  | | ⭘ ⭘ ⭘ ⭘ ⭘ | 🞎 |
| 1. Careless mistakes or slips due to a lack of attention | ⭘ | | ⭘ | | ⭘ | | ⭘ | | ⭘ | | ⭘ | | ⭘ |  | | ⭘ ⭘ ⭘ ⭘ ⭘ | 🞎 |
| 1. Gossip (including social media) | ⭘ | | ⭘ | | ⭘ | | ⭘ | | ⭘ | | ⭘ | | ⭘ |  | | ⭘ ⭘ ⭘ ⭘ ⭘ | 🞎 |
| 1. Discrimination or mobbing by another person (including social media) | ⭘ | | ⭘ | | ⭘ | | ⭘ | | ⭘ | | ⭘ | | ⭘ |  | | ⭘ ⭘ ⭘ ⭘ ⭘ | 🞎 |
| 1. Nightmares | ⭘ | | ⭘ | | ⭘ | | ⭘ | | ⭘ | | ⭘ | | ⭘ |  | | ⭘ ⭘ ⭘ ⭘ ⭘ | 🞎 |
| 1. Journey/commute to work/university/school | ⭘ | | ⭘ | | ⭘ | | ⭘ | | ⭘ | | ⭘ | | ⭘ |  | | ⭘ ⭘ ⭘ ⭘ ⭘ | 🞎 |
| 1. Minor offence (eg, fine) | ⭘ | | ⭘ | | ⭘ | | ⭘ | | ⭘ | | ⭘ | | ⭘ |  | | ⭘ ⭘ ⭘ ⭘ ⭘ | 🞎 |
| 1. Trouble with authorities, state office or other institutions (eg, tax office, bank, company) | ⭘ | | ⭘ | | ⭘ | | ⭘ | | ⭘ | | ⭘ | | ⭘ |  | | ⭘ ⭘ ⭘ ⭘ ⭘ | 🞎 |
| 1. Conflict or disagreement at work (eg, with colleagues or boss) | ⭘ | | ⭘ | | ⭘ | | ⭘ | | ⭘ | | ⭘ | | ⭘ |  | | ⭘ ⭘ ⭘ ⭘ ⭘ | 🞎 |
| 1. Conflict or disagreement **with** close persons (eg, parents, siblings, partner) | ⭘ | | ⭘ | | ⭘ | | ⭘ | | ⭘ | | ⭘ | | ⭘ |  | | ⭘ ⭘ ⭘ ⭘ ⭘ | 🞎 |
| 1. Conflict or disagreement **between** close persons (between parents, siblings, friends) | ⭘ | | ⭘ | | ⭘ | | ⭘ | | ⭘ | | ⭘ | | ⭘ |  | | ⭘ ⭘ ⭘ ⭘ ⭘ | 🞎 |
| 1. Conflict or disagreement with other non-related persons (eg, bus driver, neighbor) | ⭘ | | ⭘ | | ⭘ | | ⭘ | | ⭘ | | ⭘ | | ⭘ |  | | ⭘ ⭘ ⭘ ⭘ ⭘ | 🞎 |
| 1. Conflict or disagreement with own child/children | ⭘ | | ⭘ | | ⭘ | | ⭘ | | ⭘ | | ⭘ | | ⭘ |  | | ⭘ ⭘ ⭘ ⭘ ⭘ | 🞎 |
|  | **On approximately how many days did the situation occur?** | | | | | | | | | | | | |  | | **To what extent did you find the situation mentally straining, on average?** |  |
|  | **1** | | **2** | | **3** | | **4** | | **5** | | **6** | | **7** |  | | **Not at all Very** | **Did not occur** |
| 1. Child care problems | ⭘ | | ⭘ | | ⭘ | | ⭘ | | ⭘ | | ⭘ | | ⭘ |  | | ⭘ ⭘ ⭘ ⭘ ⭘ | 🞎 |
| 1. Running errands or transport service for other people (eg, getting medication for a family member) | | ⭘ | | ⭘ | | ⭘ | | ⭘ | | ⭘ | | ⭘ | ⭘ |  | ⭘ ⭘ ⭘ ⭘ ⭘ | | 🞎 |
| 1. Problem/inconvenience due to long distance relationships with friends/relatives | | ⭘ | | ⭘ | | ⭘ | | ⭘ | | ⭘ | | ⭘ | ⭘ |  | ⭘ ⭘ ⭘ ⭘ ⭘ | | 🞎 |
| 1. Problem/inconvenience due to a lack of help/support from others | | ⭘ | | ⭘ | | ⭘ | | ⭘ | | ⭘ | | ⭘ | ⭘ |  | ⭘ ⭘ ⭘ ⭘ ⭘ | | 🞎 |
| 1. Problem with a pet (eg, diseases, bad behavior) | | ⭘ | | ⭘ | | ⭘ | | ⭘ | | ⭘ | | ⭘ | ⭘ |  | ⭘ ⭘ ⭘ ⭘ ⭘ | | 🞎 |
| 1. Problem/inconvenience due to an unsafe environment (eg, unsafe neighborhood) | | ⭘ | | ⭘ | | ⭘ | | ⭘ | | ⭘ | | ⭘ | ⭘ |  | ⭘ ⭘ ⭘ ⭘ ⭘ | | 🞎 |
| 1. Problem/inconvenience due to dirt, pollution or smell (eg, in the neighborhood/flat) | | ⭘ | | ⭘ | | ⭘ | | ⭘ | | ⭘ | | ⭘ | ⭘ |  | ⭘ ⭘ ⭘ ⭘ ⭘ | | 🞎 |
| 1. Financial problems (not having enough money for basic services, emergencies or special wishes) | | ⭘ | | ⭘ | | ⭘ | | ⭘ | | ⭘ | | ⭘ | ⭘ |  | ⭘ ⭘ ⭘ ⭘ ⭘ | | 🞎 |
| 1. Others owe you money | | ⭘ | | ⭘ | | ⭘ | | ⭘ | | ⭘ | | ⭘ | ⭘ |  | ⭘ ⭘ ⭘ ⭘ ⭘ | | 🞎 |
| 1. You owe others money (debts) | | ⭘ | | ⭘ | | ⭘ | | ⭘ | | ⭘ | | ⭘ | ⭘ |  | ⭘ ⭘ ⭘ ⭘ ⭘ | | 🞎 |
| 1. High or unexpected financial burden (eg, purchase of expensive products, costs for a car repair) | | ⭘ | | ⭘ | | ⭘ | | ⭘ | | ⭘ | | ⭘ | ⭘ |  | ⭘ ⭘ ⭘ ⭘ ⭘ | | 🞎 |
| 1. Financial issue (eg, paying bills, planning retirement provision) | | ⭘ | | ⭘ | | ⭘ | | ⭘ | | ⭘ | | ⭘ | ⭘ |  | ⭘ ⭘ ⭘ ⭘ ⭘ | | 🞎 |
| 1. Unexpected or unwanted visit | | ⭘ | | ⭘ | | ⭘ | | ⭘ | | ⭘ | | ⭘ | ⭘ |  | ⭘ ⭘ ⭘ ⭘ ⭘ | | 🞎 |
| 1. Side effects of medications | | ⭘ | | ⭘ | | ⭘ | | ⭘ | | ⭘ | | ⭘ | ⭘ |  | ⭘ ⭘ ⭘ ⭘ ⭘ | | 🞎 |
| 1. Own physical discomfort | | ⭘ | | ⭘ | | ⭘ | | ⭘ | | ⭘ | | ⭘ | ⭘ |  | ⭘ ⭘ ⭘ ⭘ ⭘ | | 🞎 |
| 1. Physical discomfort of a close person (eg, minor illness, pain) | | ⭘ | | ⭘ | | ⭘ | | ⭘ | | ⭘ | | ⭘ | ⭘ |  | ⭘ ⭘ ⭘ ⭘ ⭘ | | 🞎 |
| 1. Lack of sleep or sleeping problems | | ⭘ | | ⭘ | | ⭘ | | ⭘ | | ⭘ | | ⭘ | ⭘ |  | ⭘ ⭘ ⭘ ⭘ ⭘ | | 🞎 |
|  | | **On approximately how many days did the situation occur?** | | | | | | | | | | | |  | **To what extent did you find the situation mentally straining, on average?** | |  |
|  | | **1** | | **2** | | **3** | | **4** | | **5** | | **6** | **7** |  | **Not at all Very** | | **Did not occur** |
| 1. Seeing a doctor | | ⭘ | | ⭘ | | ⭘ | | ⭘ | | ⭘ | | ⭘ | ⭘ |  | ⭘ ⭘ ⭘ ⭘ ⭘ | | 🞎 |
| 1. Paperwork at home (eg, filling out a form) | | ⭘ | | ⭘ | | ⭘ | | ⭘ | | ⭘ | | ⭘ | ⭘ |  | ⭘ ⭘ ⭘ ⭘ ⭘ | | 🞎 |
| 1. Housekeeping (eg, cooking, cleaning, running errands) | | ⭘ | | ⭘ | | ⭘ | | ⭘ | | ⭘ | | ⭘ | ⭘ |  | ⭘ ⭘ ⭘ ⭘ ⭘ | | 🞎 |
| 1. Minor repairs (eg, at home) | | ⭘ | | ⭘ | | ⭘ | | ⭘ | | ⭘ | | ⭘ | ⭘ |  | ⭘ ⭘ ⭘ ⭘ ⭘ | | 🞎 |
| 1. Problems with a technical device (eg, computer, household appliance, electrical device) | | ⭘ | | ⭘ | | ⭘ | | ⭘ | | ⭘ | | ⭘ | ⭘ |  | ⭘ ⭘ ⭘ ⭘ ⭘ | | 🞎 |
| 1. Maintenance (eg, of the car) | | ⭘ | | ⭘ | | ⭘ | | ⭘ | | ⭘ | | ⭘ | ⭘ |  | ⭘ ⭘ ⭘ ⭘ ⭘ | | 🞎 |
| 1. Bad weather (eg, rain, heat, cold) | | ⭘ | | ⭘ | | ⭘ | | ⭘ | | ⭘ | | ⭘ | ⭘ |  | ⭘ ⭘ ⭘ ⭘ ⭘ | | 🞎 |
| 1. Annoying behavior of misconduct of others (eg, inconsiderate smokers, annoying neighbors) | | ⭘ | | ⭘ | | ⭘ | | ⭘ | | ⭘ | | ⭘ | ⭘ |  | ⭘ ⭘ ⭘ ⭘ ⭘ | | 🞎 |
| 1. Bad food (eg, in the canteen/cafeteria) | | ⭘ | | ⭘ | | ⭘ | | ⭘ | | ⭘ | | ⭘ | ⭘ |  | ⭘ ⭘ ⭘ ⭘ ⭘ | | 🞎 |
| 1. Noise (eg, street or aircraft noise) | | ⭘ | | ⭘ | | ⭘ | | ⭘ | | ⭘ | | ⭘ | ⭘ |  | ⭘ ⭘ ⭘ ⭘ ⭘ | | 🞎 |
| 1. Traffic | | ⭘ | | ⭘ | | ⭘ | | ⭘ | | ⭘ | | ⭘ | ⭘ |  | ⭘ ⭘ ⭘ ⭘ ⭘ | | 🞎 |
| 1. Searching for a parking space | | ⭘ | | ⭘ | | ⭘ | | ⭘ | | ⭘ | | ⭘ | ⭘ |  | ⭘ ⭘ ⭘ ⭘ ⭘ | | 🞎 |
| 1. Problems with a communication medium (eg, internet, telephone) | | ⭘ | | ⭘ | | ⭘ | | ⭘ | | ⭘ | | ⭘ | ⭘ |  | ⭘ ⭘ ⭘ ⭘ ⭘ | | 🞎 |
| 1. Performance situation at work/school/university (eg, exam) | | ⭘ | | ⭘ | | ⭘ | | ⭘ | | ⭘ | | ⭘ | ⭘ |  | ⭘ ⭘ ⭘ ⭘ ⭘ | | 🞎 |
| 1. High demands/high workload at work/school/university | | ⭘ | | ⭘ | | ⭘ | | ⭘ | | ⭘ | | ⭘ | ⭘ |  | ⭘ ⭘ ⭘ ⭘ ⭘ | | 🞎 |
| 1. Boring task (eg, at work/university) | | ⭘ | | ⭘ | | ⭘ | | ⭘ | | ⭘ | | ⭘ | ⭘ |  | ⭘ ⭘ ⭘ ⭘ ⭘ | | 🞎 |
| 1. Meeting (eg, at work/university/club) | | ⭘ | | ⭘ | | ⭘ | | ⭘ | | ⭘ | | ⭘ | ⭘ |  | ⭘ ⭘ ⭘ ⭘ ⭘ | | 🞎 |
| 1. Irregular/excessively long working hours | | ⭘ | | ⭘ | | ⭘ | | ⭘ | | ⭘ | | ⭘ | ⭘ |  | ⭘ ⭘ ⭘ ⭘ ⭘ | | 🞎 |
| 1. Problem arranging and scheduling appointments | | ⭘ | | ⭘ | | ⭘ | | ⭘ | | ⭘ | | ⭘ | ⭘ |  | ⭘ ⭘ ⭘ ⭘ ⭘ | | 🞎 |
|  | | **On approximately how many days did the situation occur?** | | | | | | | | | | | |  | **To what extent did you find the situation mentally straining, on average?** | |  |
|  | | **1** | | **2** | | **3** | | **4** | | **5** | | **6** | **7** |  | **Not at all very** | | **Did not occur** |
| 1. Time pressure | | ⭘ | | ⭘ | | ⭘ | | ⭘ | | ⭘ | | ⭘ | ⭘ |  | ⭘ ⭘ ⭘ ⭘ ⭘ | | 🞎 |
| 1. Bad news (eg, rejection letter, bad grades) | | ⭘ | | ⭘ | | ⭘ | | ⭘ | | ⭘ | | ⭘ | ⭘ |  | ⭘ ⭘ ⭘ ⭘ ⭘ | | 🞎 |
| 1. Problem/inconvenience due to job/study/apprenticeship search | | ⭘ | | ⭘ | | ⭘ | | ⭘ | | ⭘ | | ⭘ | ⭘ |  | ⭘ ⭘ ⭘ ⭘ ⭘ | | 🞎 |
| 1. Problem/inconvenience due to house-hunting or moving | | ⭘ | | ⭘ | | ⭘ | | ⭘ | | ⭘ | | ⭘ | ⭘ |  | ⭘ ⭘ ⭘ ⭘ ⭘ | | 🞎 |
