# Supplementary material for: Assessment of Microstressors in Adults: Questionnaire Development and Ecological Validation of the Mainz Inventory of Microstressors
Source: JMIR Ment Health. 2020 Feb 24;7(2):e14566. doi: 10.2196/14566 (PMC7063526; doi:10.2196/14566)
Supplement: Multimedia Appendix 5 [file mental_v7i2e14566_app5.docx]

**Appendix 5: Counts and average severity of each microstressor**

**Table 6. Counts (total amount) and average severity of each microstressor over the course of four subsequent weeks (N = 70).**

| **Item** | **Assessment** | **n** | **Counts** | **Severity mean (SD)** |
| --- | --- | --- | --- | --- |
| 1. Losing or displacing objects | End-of-week | 37 | 97 | 1.26 (.97) |
|  | End-of-day | 39 | 106 | 1.68 (1.07) |
|  | EMA | 40 | 91 | 2.04 (1.08) |
| 2. Negative event in the media | End-of-week | 37 | 173 | 1.04 (.64) |
|  | End-of-day | 38 | 143 | 1.29 (.71) |
|  | EMA | 38 | 91 | 1.44 (.75) |
| 3. Negative political event | End-of-week | 31 | 160 | 1.28 (.69) |
|  | End-of-day | 31 | 116 | 1.47 (.67) |
|  | EMA | 28 | 74 | 2.09 (.76) |
| 4. Social obligation | End-of-week | 47 | 337 | 1.16 (.72) |
|  | End-of-day | 51 | 345 | 1.15 (.86) |
|  | EMA | 46 | 292 | 1.34 (.83) |
| 5. Interruption during an activity (eg, at work or during leisure activities) | End-of-week | 62 | 693 | 1.11 (.82) |
|  | End-of-day | 63 | 717 | 1.15 (.71) |
|  | EMA | 64 | 642 | 1.46 (.81) |
| 6. Waiting time or delay (eg, waiting for a person; bus or train delay) | End-of-week | 68 | 741 | 1.23 (.75) |
|  | End-of-day | 67 | 774 | 1.32 (.71) |
|  | EMA | 70 | 701 | 1.51 (.79) |
| 7. Careless mistakes or slips due to a lack of attention | End-of-week | 37 | 226 | 1.50 (.93) |
|  | End-of-day | 46 | 253 | 1.54 (.87) |
|  | EMA | 47 | 207 | 1.87 (.81) |
| 8. Gossip (including social media) | End-of-week | 16 | 31 | 1.59 (.99) |
|  | End-of-day | 20 | 38 | 1.24 (.85) |
|  | EMA | 16 | 31 | 2.18 (1.09) |
| 9. Discrimination or mobbing by another person (including social media) | End-of-week | 1 | 3 | 3.00 (-) |
|  | End-of-day | 6 | 6 | 1.00 (1.10) |
|  | EMA | 6 | 9 | 1.21 (1.15) |
| 10. Nightmares | End-of-week | 39 | 145 | 1.68 (.92) |
|  | End-of-day | 40 | 114 | 1.64 (.89) |
|  | EMA | 41 | 113 | 1.86 (.79) |
| 11. Journey/commute to work/ university/ school | End-of-week | 65 | 987 | .98 (.80) |
|  | End-of-day | 66 | 920 | .92 (.65) |
|  | EMA | 65 | 799 | 1.09 (.80) |
| 12. Minor offence (eg, fine) | End-of-week | 7 | 12 | .93 (1.17) |
|  | End-of-day | 10 | 11 | 1.2 (1.23) |
|  | EMA | 4 | 4 | 2.75 (.96) |
| 13. Trouble with authorities, state office or other institutions (eg, tax office, bank, company) | End-of-week | 22 | 67 | 1.97 (.86) |
|  | End-of-day | 30 | 80 | 1.69 (.88) |
|  | EMA | 28 | 62 | 2.21 (1.31) |
| 14. Conflict or disagreement at work (eg, with colleagues or boss) | End-of-week | 24 | 69 | 1.90 (.87) |
|  | End-of-day | 26 | 77 | 1.77 (.93) |
|  | EMA | 28 | 66 | 2.20 (.93) |
| 15. Conflict or disagreement **with** close persons (eg, parents, siblings, partner) | End-of-week | 51 | 266 | 2.19 (.90) |
|  | End-of-day | 52 | 257 | 1.99 (.94) |
|  | EMA | 51 | 208 | 2.43 (.75) |
| 16. Conflict or disagreement **between** close persons (between parents, siblings, friends) | End-of-week | 20 | 43 | 1.72 (1.03) |
|  | End-of-day | 27 | 43 | 1.73 (1.06) |
|  | EMA | 32 | 51 | 1.82 (1.00) |
| 17. Conflict or disagreement with other non-related persons (eg, bus driver, neighbor) | End-of-week | 22 | 44 | 1.44 (.89) |
|  | End-of-day | 26 | 59 | 1.80 (.83) |
|  | EMA | 32 | 63 | 2.10 (.83) |
| 18. Conflict or disagreement with own child/children | End-of-week | 3 | 33 | 1.94 (.73) |
|  | End-of-day | 5 | 39 | .97 (.97) |
|  | EMA | 3 | 32 | 2.06 (.64) |
| 19. Child care problems | End-of-week | 4 | 14 | 2.00 (.00) |
|  | End-of-day | 7 | 22 | 1.07 (.79) |
|  | EMA | 5 | 13 | 1.85 (.42) |
| 20. Running errands or transport service for other people (eg, getting medication for a family member) | End-of-week | 21 | 50 | 1.12 (.79) |
|  | End-of-day | 23 | 52 | 1.03 (.73) |
|  | EMA | 22 | 52 | 1.06 (.87) |
| 21. Problem/inconvenience due to long distance relationships with friends/relatives | End-of-week | 46 | 249 | 1.57 (1.07) |
|  | End-of-day | 47 | 237 | 1.65 (1.04) |
|  | EMA | 37 | 118 | 2.11 (.93) |
| 22. Problem/inconvenience due to a lack of help/support from others | End-of-week | 23 | 75 | 1.54 (.75) |
|  | End-of-day | 25 | 87 | 1.67 (.76) |
|  | EMA | 27 | 56 | 2.16 (.82) |
| 23. Problem with a pet (eg, diseases, bad behavior) | End-of-week | 8 | 39 | 2.40 (1.01) |
|  | End-of-day | 12 | 37 | 1.55 (.91) |
|  | EMA | 8 | 23 | 2.01 (1.34) |
| 24. Problem/inconvenience due to an unsafe environment (eg, unsafe neighborhood) | End-of-week | 9 | 69 | 1.50 (1.06) |
|  | End-of-day | 9 | 12 | 1.20 (.89) |
|  | EMA | 7 | 9 | 1.96 (.82) |
| 25. Problem/inconvenience due to dirt, pollution or smell (eg, in the neighborhood/flat) | End-of-week | 26 | 77 | 1.41 (.76) |
|  | End-of-day | 32 | 89 | 1.21 (.70) |
|  | EMA | 28 | 67 | 1.81 (.80) |
| 26. Financial problems (not having enough money for basic services, emergencies or special wishes) | End-of-week | 16 | 70 | 1.94 (1.18) |
|  | End-of-day | 18 | 49 | 1.57 (.96) |
|  | EMA | 16 | 30 | 2.49 (1.04) |
| 27. Others owe you money | End-of-week | 14 | 65 | .75 (.73) |
|  | End-of-day | 20 | 68 | .84 (.82) |
|  | EMA | 11 | 19 | 1.56 (1.15) |
| 28. You owe others money (debts) | End-of-week | 19 | 57 | 1.12 (1.12) |
|  | End-of-day | 23 | 52 | 1.14 (.99) |
|  | EMA | 16 | 26 | 1.40 (1.33) |
| 29. High or unexpected financial burden (eg, purchase of expensive products, costs for a car repair) | End-of-week | 26 | 76 | 1.58 (1.09) |
|  | End-of-day | 29 | 71 | 1.33 (.82) |
|  | EMA | 19 | 56 | 2.05 (.96) |
| 30. Financial issue (eg, paying bills, planning retirement provision) | End-of-week | 27 | 120 | 1.13 (.89) |
|  | End-of-day | 36 | 141 | 1.35 (.84) |
|  | EMA | 39 | 117 | 1.56 (.77) |
| 31. Unexpected or unwanted visit | End-of-week | 28 | 67 | .68 (.77) |
|  | End-of-day | 30 | 84 | .98 (.99) |
|  | EMA | 29 | 57 | 1.31 (1.11) |
| 32. Side effects of medications | End-of-week | 8 | 28 | 2.16 (.93) |
|  | End-of-day | 13 | 28 | 1.64 (1.04) |
|  | EMA | 9 | 19 | 2.20 (1.37) |
| 33. Own physical discomfort | End-of-week | 67 | 600 | 1.82 (.77) |
|  | End-of-day | 64 | 649 | 1.63 (.65) |
|  | EMA | 65 | 517 | 1.97 (.69) |
| 34. Physical discomfort of a close person (eg, minor illness, pain) | End-of-week | 25 | 102 | 1.81 (1.00) |
|  | End-of-day | 38 | 122 | 1.67 (.88) |
|  | EMA | 37 | 106 | 1.96 (.98) |
| 35. Lack of sleep or sleeping problems | End-of-week | 66 | 603 | 1.79 (.76) |
|  | End-of-day | 66 | 650 | 1.63 (.70) |
|  | EMA | 62 | 544 | 1.98 (.68) |
| 36. Seeing a doctor | End-of-week | 18 | 42 | .90 (1.05) |
|  | End-of-day | 23 | 44 | .94 (.67) |
|  | EMA | 19 | 41 | 1.12 (.94) |
| 37. Paperwork at home (eg, filling out a form) | End-of-week | 45 | 348 | 1.12 (.83) |
|  | End-of-day | 51 | 353 | 1.22 (.74) |
|  | EMA | 47 | 263 | 1.26 (.83) |
| 38. Housekeeping (eg, cooking, cleaning, running errands) | End-of-week | 66 | 982 | .67 (.66) |
|  | End-of-day | 65 | 966 | .71 (.63) |
|  | EMA | 64 | 756 | .88 (.78) |
| 39. Minor repairs (eg, at home) | End-of-week | 21 | 39 | .47 (.74) |
|  | End-of-day | 28 | 54 | 1.00 (1.09) |
|  | EMA | 18 | 33 | 1.10 (.97) |
| 40. Problems with a technical device (eg, computer, household appliance, electrical device) | End-of-week | 42 | 174 | 1.63 (1.01) |
|  | End-of-day | 51 | 206 | 1.50 (.85) |
|  | EMA | 42 | 144 | 1.91 (1.00) |
| 41. Maintenance (eg, of the car) | End-of-week | 15 | 22 | 1.03 (.85) |
|  | End-of-day | 25 | 31 | 1.36 (.99) |
|  | EMA | 17 | 17 | 1.69 (1.23) |
| 42. Bad weather (eg, rain, heat, cold) | End-of-week | 60 | 429 | 1.17 (.72) |
|  | End-of-day | 62 | 487 | 1.35 (.72) |
|  | EMA | 58 | 312 | 1.55 (.80) |
| 43. Annoying behavior of misconduct of others (eg, inconsiderate smokers, annoying neighbors) | End-of-week | 32 | 103 | 1.77 (1.00) |
|  | End-of-day | 44 | 104 | 1.71 (.93) |
|  | EMA | 37 | 97 | 2.05 (.85) |
| 44. Bad food (eg, in the canteen/cafeteria) | End-of-week | 21 | 55 | 1.24 (.85) |
|  | End-of-day | 32 | 69 | 1.11 (.70) |
|  | EMA | 31 | 63 | 1.64 (.80) |
| 45. Noise (eg, street or aircraft noise) | End-of-week | 29 | 116 | 1.26 (1.03) |
|  | End-of-day | 40 | 127 | 1.11 (.88) |
|  | EMA | 38 | 111 | 1.57 (.79) |
| 46. Traffic | End-of-week | 34 | 142 | 1.46 (.81) |
|  | End-of-day | 40 | 142 | 1.30 (.77) |
|  | EMA | 42 | 114 | 1.85 (1) |
| 47. Searching for a parking space | End-of-week | 31 | 141 | .98 (.96) |
|  | End-of-day | 36 | 144 | 1.12 (1.00) |
|  | EMA | 25 | 101 | 1.49 (1.03) |
| 48. Problems with a communication medium (eg, internet, telephone) | End-of-week | 37 | 194 | 1.70 (.92) |
|  | End-of-day | 44 | 178 | 1.60 (.91) |
|  | EMA | 46 | 95 | 2.03 (.85) |
| 49. Performance situation at work/school/university (eg, exam) | End-of-week | 42 | 305 | 2.17 (1.00) |
|  | End-of-day | 46 | 269 | 2.16 (.82) |
|  | EMA | 41 | 207 | 2.38 (.94) |
| 50. High demands/high workload at work/school/university | End-of-week | 54 | 670 | 2.15 (.75) |
|  | End-of-day | 59 | 611 | 2.06 (.71) |
|  | EMA | 57 | 510 | 2.24 (.76) |
| 51. Boring task (eg, at work/university) | End-of-week | 56 | 459 | 1.26 (.78) |
|  | End-of-day | 59 | 471 | 1.28 (.68) |
|  | EMA | 62 | 457 | 1.49 (.76) |
| 52. Meeting (eg, at work/university/club) | End-of-week | 48 | 211 | .79 (.79) |
|  | End-of-day | 51 | 222 | 1.05 (.86) |
|  | EMA | 45 | 161 | 1.15 (.89) |
| 53. Irregular/excessively long working hours | End-of-week | 36 | 168 | 1.76 (.97) |
|  | End-of-day | 41 | 164 | 1.71 (.78) |
|  | EMA | 33 | 98 | 1.97 (.76) |
| 54. Problem arranging and scheduling appointments | End-of-week | 46 | 235 | 1.64 (.91) |
|  | End-of-day | 51 | 260 | 1.67 (.79) |
|  | EMA | 53 | 207 | 1.89 (.79) |
| 55. Time pressure | End-of-week | 59 | 666 | 1.98 (.82) |
|  | End-of-day | 65 | 737 | 1.77 (.69) |
|  | EMA | 67 | 673 | 2.06 (.68) |
| 56. Bad news (eg, rejection letter, bad grades) | End-of-week | 22 | 51 | 2.02 (1.37) |
|  | End-of-day | 29 | 65 | 1.99 (.92) |
|  | EMA | 33 | 66 | 2.23 (1.00) |
| 57. Problem/inconvenience due to job/study/apprenticeship search | End-of-week | 6 | 18 | 1.86 (1.15) |
|  | End-of-day | 12 | 37 | 1.54 (.95) |
|  | EMA | 11 | 31 | 2.57 (.76) |
| 58. Problem/inconvenience due to house-hunting or moving | End-of-week | 9 | 33 | 2.00 (.87) |
|  | End-of-day | 12 | 38 | 2.30 (.81) |
|  | EMA | 13 | 29 | 2.41 (.76) |

**Notes:** SD = standard deviation; in order to compare the microstressor data reported in the end-of-week, end-of-day and EMA assessments, data from the EMA or end-of-day assessments were aggregated, so that microstressors reported several times a day were counted only once. Counts were then summed across the entire study sample. Thus, with a maximum of 28 possible counts per stressor (from end-of-week, end-of-day or EMA assessments) in a sample with *n* = 70 subjects, the maximum number of summed counts per microstressor is 1960. This means that the two most frequent stressors (commuting, housekeeping) were reported on average every other day. For all of the microstressors the summed counts per microstressor in the end-of-week and end-of-day assessments were higher than in the EMA assessment. Note that the response rates varied between the three assessments modalities; the ten most frequent microstressors (counts, referring to end-of-week assessment): 11, 38, 6, 5, 50, 55, 35, 33, 51, 42; the ten most severe microstressors (referring to end-of-week assessment): 9, 23, 15, 49, 32, 50, 56, 19, 58, 55.
